# Global discovery and characterization of small non-coding RNAs in marine microalgae

**DOI:** 10.1186/1471-2164-15-697

**Published:** 2014-08-20

**Authors:** Sara Lopez-Gomollon, Matthew Beckers, Tina Rathjen, Simon Moxon, Florian Maumus, Irina Mohorianu, Vincent Moulton, Tamas Dalmay, Thomas Mock

**Affiliations:** School of Biological Sciences, University of East Anglia, Norwich, NR4 7TJ UK; School of Computing Sciences, University of East Anglia, Norwich, NR4 7TJ UK; UR1164 URGI-Research Unit in Genomics-Info, INRA de Versailles-Grignon, Route de Saint-Cyr, Versailles, 78026 France; School of Environmental Sciences, University of East Anglia, Norwich, NR4 7TJ UK; Estación Experimental Aula Dei, CSIC (Consejo Superior de Investigaciones Científicas), 50059 Zaragoza, Spain; Commonwealth Scientific and Industrial Research Organization Plant Industry, Canberra, Australian Capital Territory, 2601 Australia; The Genome Analysis Centre, Norwich, NR4 7UH UK

**Keywords:** Coccolithophores, Diatoms, Growth, Marine phytoplankton, MicroRNA, Non-coding RNAs, Small RNA, Stress, tRNA

## Abstract

**Background:**

Marine phytoplankton are responsible for 50% of the CO_2_ that is fixed annually worldwide and contribute massively to other biogeochemical cycles in the oceans. Diatoms and coccolithophores play a significant role as the base of the marine food web and they sequester carbon due to their ability to form blooms and to biomineralise. To discover the presence and regulation of short non-coding RNAs (sRNAs) in these two important phytoplankton groups, we sequenced short RNA transcriptomes of two diatom species (*Thalassiosira pseudonana*, *Fragilariopsis cylindrus*) and validated them by Northern blots along with the coccolithophore *Emiliania huxleyi*.

**Results:**

Despite an exhaustive search, we did not find canonical miRNAs in diatoms. The most prominent classes of sRNAs in diatoms were repeat-associated sRNAs and tRNA-derived sRNAs. The latter were also present in *E. huxleyi*. tRNA-derived sRNAs in diatoms were induced under important environmental stress conditions (iron and silicate limitation, oxidative stress, alkaline pH), and they were very abundant especially in the polar diatom *F. cylindrus* (20.7% of all sRNAs) even under optimal growth conditions.

**Conclusions:**

This study provides first experimental evidence for the existence of short non-coding RNAs in marine microalgae. Our data suggest that canonical miRNAs are absent from diatoms. However, the group of tRNA-derived sRNAs seems to be very prominent in diatoms and coccolithophores and maybe used for acclimation to environmental conditions.

**Electronic supplementary material:**

The online version of this article (doi:10.1186/1471-2164-15-697) contains supplementary material, which is available to authorized users.

## Background

Marine phytoplankton are responsible for ca. 50% of the CO_2_ that is fixed annually, worldwide and therefore vital for climate control [1]. This is especially true for eukaryotic phytoplankton (microalgae) such as diatoms (stramenopiles) and coccolithophores (haptophytes), which make a large impact on biogeochemical cycles because of their “bloom and bust” lifestyle [2] and their ability to biomineralise. This makes them key organisms for carbon sequestration [3, 4] but also targets for bio-nanotechnology research [5, 6]. Furthermore, these microalgae are the base of the marine food web [1] and therefore fulfil important ecosystem services and contribute to food security (e.g. fisheries) similar to the ecological role of plants in terrestrial ecosystems.

The first genome sequences from diatoms [7–9] and coccolithophores [10] have only recently become available and the post-genomics era for marine microalgae has just begun [11, 12]. Unlike green algae, red algae, and plants, diatoms and coccolithophores have evolved from secondary endosymbiosis events whereby a eukaryotic unicellular heterotroph acquired a plastid through enslavement of ancestral red and/or green algae [13, 14]. One consequence of these endosymbiotic events was a massive horizontal gene transfer from the endosymbionts towards host genomes, which is detectable in the genomes of all sequenced diatom and coccolithophore species [7–10]. Furthermore, coccolithophore and especially diatom genomes have been significantly shaped by horizontal gene transfer from Bacteria and Archaea [7–10]. How this mosaic of genes, with different evolutionary histories, is regulated to orchestrate metabolic responses to environmental conditions is still largely unknown.

The surface ocean is subject to both dynamic changes on a short time scale and changes caused by global warming and ocean acidification [15]. Thus, marine microalgae must have evolved mechanisms that regulate gene activity [11, 12] to be able to differentially respond to a significant variety of environmental stresses in the surface oceans.

An organism’s response to environmental conditions consists of altering levels of gene expression, which in turn leads to a phenotypic change in the organism [11]. Various transcriptional and post-transcriptional processes govern transcript levels. Several of these mechanisms are based on 15–30 nucleotide short non-coding RNAs (sRNAs) that are produced through different pathways and target either mRNA or genomic DNA through sequence complementarity [16]. Major groups of sRNAs include microRNAs (miRNAs) and short-interfering RNAs (siRNAs) [17, 18]. miRNAs are processed from non-coding genes that produce single stranded precursors with relatively stable hairpin secondary structures that are processed into short, mature miRNAs by Dicer-like proteins. When loaded onto multi-protein complexes (e.g. RISC), miRNAs can target and downregulate mRNAs based on sequence complementarity. siRNAs are generated from long double-stranded RNAs that are cut by Dicer-like proteins and then incorporated into protein complexes that either target complementary mRNA for cleavage or genomic DNA for epigenetic modification (e.g. DNA methylation) [19].

sRNAs corresponding to fragments of rRNAs, tRNAs, snoRNAs, and snRNAs are also highly abundant in sRNA deep sequencing data [20]. There is mounting evidence that some of these sRNAs, long thought to be degradation products, are actually the products of processing events, with an associated biological function. tRNA fragments, for example, have been found in a diverse range of organisms, and can be categorised into two distinct classes: tRNA-halves, which are 35-40 nt in length, arise from a cleavage of the anticodon loop and are upregulated under cellular stress [21, 22], and shorter tRNA sRNAs (tsRNAs) which arise from cleavage in the left or right arm of the tRNA, leaving a sRNA of similar size to a miRNA at around 16-23 nt in length [22–24]. The function of these tRNA fragments, as well as many of the other RNA fragments, remains up for debate. This extended class of tsRNAs are an important consideration in any sRNA sequencing project due to the ease in which they can be mistaken for miRNAs. For example, due to the presence of hairpin structures in tRNAs and other non-coding RNAs, these are often predicted as precursor miRNAs by prediction programs [25].

The universal presence of sRNAs in multicellular eukaryotes has triggered the question about their origin. First reports of miRNAs, a group of best-studied sRNAs, in the freshwater green alga *Chlamydomonas reinhardtii* revealed a more ancient origin of sRNAs and gave evidence of their presence also in unicellular organisms [26]. Very recently, the first sRNA transcriptomes from two marine diatoms have been published, which provide hints for the existence of miRNAs in marine eukaryotic algae [27, 28]. However, neither of these two studies has provided experimental evidence for their existence, which leaves the question as to whether marine algae such as diatoms and coccolithophores, which are evolutionary very distantly related to other eukaryotes, possess miRNAs.

In this study, we used a comprehensive molecular approach to detect and to experimentally validate sRNAs in two different diatoms (*Thalassiosira pseudonana, Fragilariopsis cylindrus*) and a coccolithophore (*Emiliania huxleyi*). Our study, based on Illumina sequencing and Northern blot analysis of sRNAs, revealed that diatoms most likely do not possess canonical miRNAs as we know them from other organisms. However, diatoms seem to have repeat-associated sRNAs and all microalgae tested in our study possess tRNA-derived sRNAs that were differentially regulated depending on the stress condition applied. Thus, our results provide the first evidence of the existence of sRNAs in diatoms and coccolithophores and their role for coping with important environmental conditions of the surface oceans.

## Results

### Short RNAs from diatoms (*Thalassiosira pseudonana*, *Fragilariopsis cylindrus*)

#### miRNA analysis

miRNAs were recently predicted to exist in diatoms [27, 28]. However, none of these studies validated any of the predicted miRNAs by experimental approaches. To assess the existence of miRNAs in diatoms, we focussed on the model diatoms *T. pseudonana* and *F. cylindrus* and undertook a dual approach. The first and early approach was targeted exclusively on potential miRNAs in *T. pseudonana*, and the second and more comprehensive approach included an analysis of all potential classes of sRNAs in *T. pseudonana* and *F. cylindrus*.

For the first approach, RNA from *T. pseudonana* grown under different environmental conditions (silicon- and iron- limitation as well as alkaline pH, which decreases dissolved CO_2_ concentration) [11] was used to obtain cDNA libraries of sRNAs. The libraries were subjected to deep sequencing on the Illumina GAII platform. In combination, the bioinformatic tool miRCat [29] was used for *de novo* miRNA prediction from the whole genome sequence. At first, the predicted miRNAs with differential read numbers in the different samples were investigated. Thirty-two such potential miRNAs were checked by sRNA Northern blotting with their corresponding positive controls, but none of them were detectable. As the read numbers of these sRNAs were relatively low, we also tested another set of potential miRNAs that had high read numbers but were not differentially expressed during abiotic stress (19 sRNAs checked). However, these sequences were not detectable either (data not shown). To rule out the possibility that the concentration of sRNAs in diatoms were so low that it was below the limit of detection, the sRNA fraction was purified from 780 μg total RNA, but even after using 150 times more RNA than necessary to detect miRNAs in other organisms [30] we were unable to detect any of these predicted miRNAs. It is worth mentioning that the protocol used for cDNA library generation (see Methods section) involved isolating the miRNA-like size fraction from total RNA to enrich for the sequences of 19–24 nt length and also purifying the ligation product from gel after each adapter ligation, therefore potential products shorter or longer than 19–24 nt were missed in this first approach.

**Figure 1:**
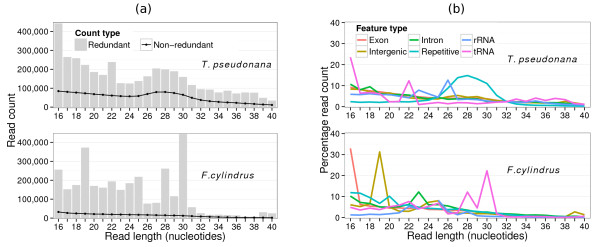
**Global size class distribution reads of** ***T. pseudonana*** **and** ***F. cylindrus.*** Global size class distributions of sequences mapping to the genome, excluding rRNA sequences **(a)** and sequences mapping to selected features, counted as a proportion of the sequences mapping to a particular feature **(b)** of *T. pseudonana* and *F. cylindrus.*

For the second and more comprehensive approach, we generated new sRNA libraries from the diatoms *T. pseudonana* and also from *F. cylindrus,* a polar psychrophilic diatom species [31]. The methodology to obtain the libraries had improved so it was not necessary to purify sRNAs or ligation products from gel. The small RNA v1.5 kit (Illumina) did not involve any gel purification until the PCR step, therefore it was possible to see bands containing a sequence outside the 19–24 nt range. We noticed that the band corresponding to the library for *T. pseudonana* was wider than expected when compared to libraries from animal or plant sRNAs. The predominant size distribution corresponded to a mixed population of sRNAs of 25–35 nt (Additional file 1: Figure S1). This result suggested that our previous libraries may not have been comprehensive because the gel purification steps enriched the library in sequences of size between 19–24 nt, so the majority of sRNAs from *T. pseudonana* were not included. We obtained 61,572,264 and 39,250,410 total reads from *T. pseudonana* and *F. cylindrus*, respectively after removing adapter sequences (Table 1). Size class distributions were obtained for sequences that matched the corresponding genome with no mismatches (Figure 1a). These distributions did not include sequences from rRNA sources, since they produced a large amount of degradation product which obscured size class patterns in other features. Highly abundant size classes can indicate the presence of sRNAs of a certain length. In particular, miRNAs form a 21 nt and 22 nt peak in plants and animals, respectively, and the heterochromatic siRNAs yield a 24 nt peak in plants. However, neither diatom sRNA library showed a defined peak for these size classes. Nevertheless, we analysed our sRNA transcriptomes to find potential miRNAs and tried to validate them by Northern blotting. The miRNA prediction tools miRCat [32] and miRDeep2 [33] were both run on the sequences for *T. pseudonana* and *F. cylindrus.* In total, 46 mature miRNA candidates in *T. pseudonana* and 177 candidates in *F. cylindrus* were identified by computational analysis. Interestingly, there were no candidates that both prediction tools (miRCat, MiRDeep2) could agree on because the two prediction programs use different criteria for miRNA classification (Additional file 1: Figure S2). In addition, none of the candidates identified in *T. pseudonana* matched miRNA candidates found in the same species in a previous study [27].

**Table 1:** **Read counts in** ***T. pseudonana*** **and** ***F. cylindrus*** **sRNA libraries**

Sequence reads	*T. pseudonana*		*F. cylindrus*	
	Redundant	Unique	Redundant	Unique
Total reads	1.08 × 10^08^	NA	1.0 × 10^08^	NA
Adapter removal	61572264	5158180	39250410	1837242
After mapping	39033362	1432106	28829893	430009
Percentage (%) mapped	63	28	47	8

Summary of read counts in the sRNA libraries at each stage of pre-processing and mapping to transcripts.

Seven candidates (one from *T. pseudonana* and six from *F. cylindrus*) had between 321 and 10643 read counts. The rest of the candidates were below 100 read counts. The seven most abundant candidates all mapped to loci that were annotated as tRNA or rRNA except Fc-2 (Additional file 2), although some also mapped to other loci that were not annotated. The only *T. pseudonana* predicted miRNA with a high read number was also found to map to a repetitive and abundant rRNA locus (see Methods section).

**Figure 2:**
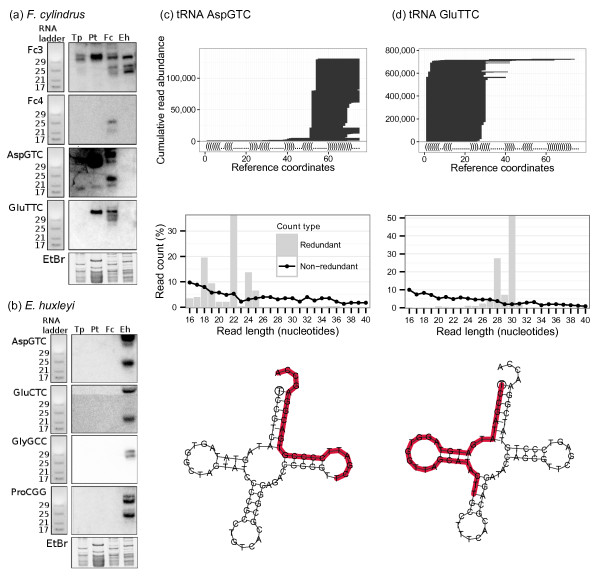
**sRNAs from** ***F. cylindrus*** **and** ***E. huxleyi.*** Northern blot validation of *F. cylindrus* **(a)** and *E. huxleyi* **(b)** candidates and their expression in *T. pseudonana* (Tp), *P. tricornutum* (Pt), *F. cylindrus* (Fc), and *E. huxleyi* (Eh). ZR small RNA ladder (left) and EtBr gel (bottom) are provided for band sizing and to show equal loading of the above RNAs. **(c)** and **(d)**, examples of *F. cylindrus* validated tRNAs producing sRNA sequences. The top visualisation represents the mapping pattern of sRNAs stacked up against the y-axis mapped to the tRNA sequence on the x-axis. The secondary structure of the tRNA is shown in dot-bracket notation above the x-axis. The middle graph shows the size class distribution of all sRNAs that map to the tRNA and the bottom schematic shows the folded tRNA with its most abundant sRNA from the library highlighted in red.

We assessed these most abundant candidates in *F. cylindrus* by Northern blot but only two probes (Fc-3 and Fc-4) gave a positive result (Figure 2a). Both probes hybridised to more than one band, which is uncharacteristic of miRNAs, and the patterns were similar to blots from tRNA-derived reads (Figure 2a). It was not surprising since Fc-3 maps to a tRNA SerTGA locus and Fc-4 maps to a tRNA GluTTC locus. Fc-4 sequence maps to a few intergenic regions with potential stem-loop structure, but since it is an exact 3’ end sequence of a tRNA we do not consider it as a putative miRNA. In summary, none of the predicted miRNAs were validated from separate sRNA libraries of two different diatoms suggesting that canonical miRNAs most likely do not exist in diatoms.

#### Short RNAs from repetitive elements

Our approach for identifying any kind of sRNAs revealed enrichment of other size classes in both diatom species. Size classes including sequences that were less than 18 nt long and between 26 and 30 nt in *T. pseudonana* were enriched, which was in agreement with the sRNA library band size obtained in the polyacrylamide gel (Additional file 1: Figure S1). In *F. cylindrus*, specific size classes of 19 nt, 28 nt and 32 nt were enriched (Figure 1a). To assess the origins of as many sRNA sequences as possible in both diatom genomes, an annotation pipeline was created that would map sequences to various transcripts and features (Figure 1b). Repetitive elements are an interesting source of sRNAs, especially in *T. pseudonana*, where 22% of sRNAs mapping to them. Unexpectedly, sRNAs that mapped to repetitive elements in this diatom showed a distinct size class of 26-30 nt, which is unusual for putative siRNAs. These sequences also showed a significant bias towards uracil at their 5’ ends (Additional file 1: Figure S3). The bias is similar to those shown by miRNAs that associate with Argonaute [34]. In contrast, fewer sequences mapped to repetitive elements in *F. cylindrus* and these did not show any specific nucleotide bias or size class enrichments.

#### Transfer RNA-derived short RNAs

**Figure 3:**
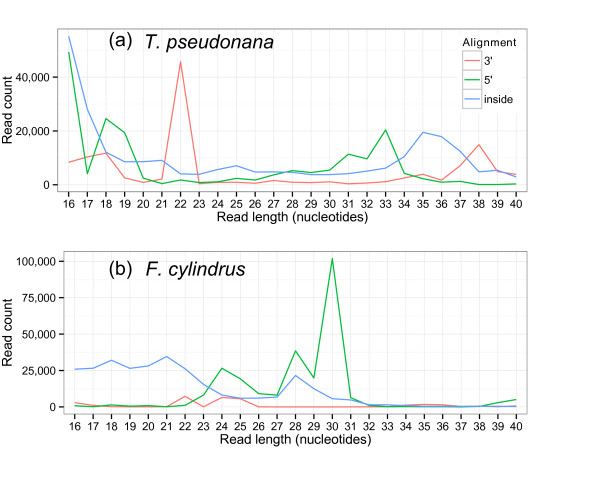
**Size class distributions of reads that map to mature tRNA transcripts for a)** ***T. pseudonana*** **and b)** ***F. cylindrus*** **.** Alignment of a size distribution of mapped reads of sRNA to their tRNAs. The reads are grouped by the type of match: 5’ end, between ends or 3’ ends of the tRNA.

**Figure 4:**
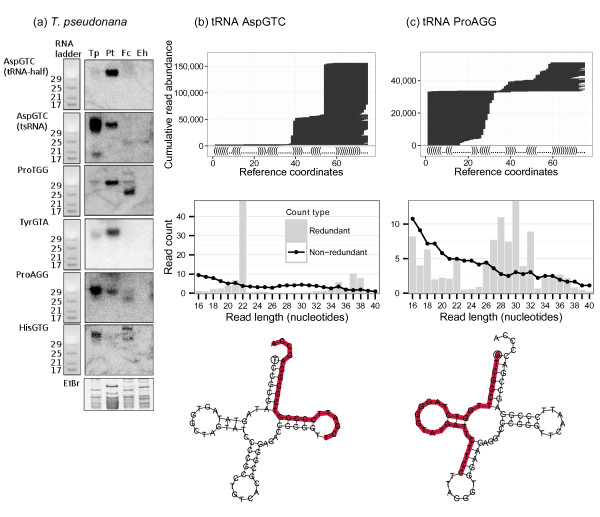
**tRNA-derived sRNAs in** ***T. pseudonana.*** Northern blots in **(a)** show validation of selected sequences from different tRNAs in *T. pseudonana* and their expression in *T. pseudonana* (Tp), *P. tricornutum* (Pt), *F. cylindrus* (Fc), and *E. huxleyi* (Eh). ZR small RNA ladder on the left of each blot is shown to size the bands and EtBr is used for equal loading. Examples of two tRNAs expressing sRNAs within the *T. pseudonana* sequenced libraries are shown in **(b)** and **(c)**. The top visualisation represents the mapping pattern of sRNAs stacked up against the y-axis mapped to the tRNA sequence on the x-axis. The secondary structure of the tRNA is shown in dot-bracket notation above the x-axis. The middle graph shows the size class distribution of all sRNAs that map to the tRNA and the bottom schematic shows the folded tRNA with its most abundant sRNA from the library highlighted in red.

In *F. cylindrus*, 1,865,089 sequences (20.7% of mapped sequences) mapped to tRNA loci, compared with 886,908 sequences (2.9%) in *T. pseudonana* (Additional file 3). Furthermore, the tRNA-derived sRNAs were able to explain the specific size class enrichments in *F. cylindrus* at 28 nt and 32 nt. Thus, tRNA-derived sRNAs were the dominant group of sRNAs in *F. cylindrus* and they were also very abundant in *T. pseudonana*. The abundance of sRNAs that derive from particular tRNA types in our study was not uniform, and the abundance of sequences from all shared tRNA types between the two diatoms correlated with a Pearson correlation coefficient of 0.411 (P = 0.009, where the null hypothesis is that the compared distributions have no correlation) (Additional file 1: Figure S4a). The most abundant tsRNA reads for both diatoms were derived from AspGTC, GluCTC, and HisGTG tRNAs, with GluTTC as an outlier which was abundant only in *F. cylindrus*. Another large difference between the tRNA-derived reads of the two diatoms were the cleavage patterns made along the tRNA structure. Reads that aligned precisely to the ends of tRNAs were cleaved within a bulge 92% of the time in *T. pseudonana* but only 21% of the time in *F. cylindrus*, the remaining cleavage occurring within the base-paired parts of the tRNA. The general consensus for tsRNA and tRNA-halves is that they are cleaved within the loop of a specific tRNA [22–24]. Although the tRNAs that produce most of the sRNAs in our data appeared to be consistent between the two species, the nature of the sRNAs was not (Additional file 1: Figure S4b). *T. pseudonana* tRNAs showed a preference for producing tsRNAs from the 3’ end, whereas *F. cylindrus* tRNAs had a preference for 5’ tRNA-halves (Figure 3). These preferences were only clear for sRNAs that mapped precisely to the tRNA ends. Few abundant tRNA-derived sRNAs were shared between the diatoms as their conservation depended entirely on the conservation of the precursor tRNAs. However, one tRNA, AspGTC, was consistent in producing abundant sRNAs in both diatoms that was also validated by Northern blot (Figures 2c and 4b).

Six tRNA-derived sRNAs were validated in *T. pseudonana*, five of which were conserved in *P. tricornutum* and four in *F. cylindrus* but none of them were present in *E. huxleyi* (Figure 4a)*.*

In the case of *F. cylindrus*, we identified three sequences that originated from tRNAs and two of them were validated. The sRNA mapping to tRNA GluTTC locus gave a pattern of 4 bands of sizes between 25–31 nt, and is conserved only in *P. tricornutum*. The sRNA derived from tRNA AspGTC was conserved in *P. tricornutum* and *T. pseudonana* but not in *E. huxleyi* (Figure 2a).

### Short RNAs from coccolithophores (*Emiliania huxleyi*)

For *E. huxleyi*, we did not sequence sRNAs. However, in order to look for the presence of sRNAs generated from tRNAs in this organism, we identified tRNAs in the genome of the *E. huxleyi* CCMP1516 strain and tried probes from four different tRNAs at different positions. Only one of the tRNA-halves found in *T. pseudonana* or *F. cylindrus* was found in *E. huxleyi* based on sequence similarity. In order to look for the presence of other tRNA-derived sRNAs in *E. huxley*i, we designed several probes complementary to predicted tRNAs that were tested by Northern blotting. Four of them were positive and quite specific to *E. huxleyi* as they were not detected in any of the other three diatoms tested. One of them (GlyGCC) only produced tRNA-halves, but the other three generated tsRNAs as well (Figure 2b).

### Transfer RNA derived short RNAs are upregulated by abiotic stress

One of the main mechanisms to cope with stress (e.g. nutrient limitation) involves an increase in the production of stress response protein when translation is generally repressed [35]. Recently, it has been proposed that tRNAs not only play a fundamental role in translation but also act to regulate cell response [36].

**Figure 5:**
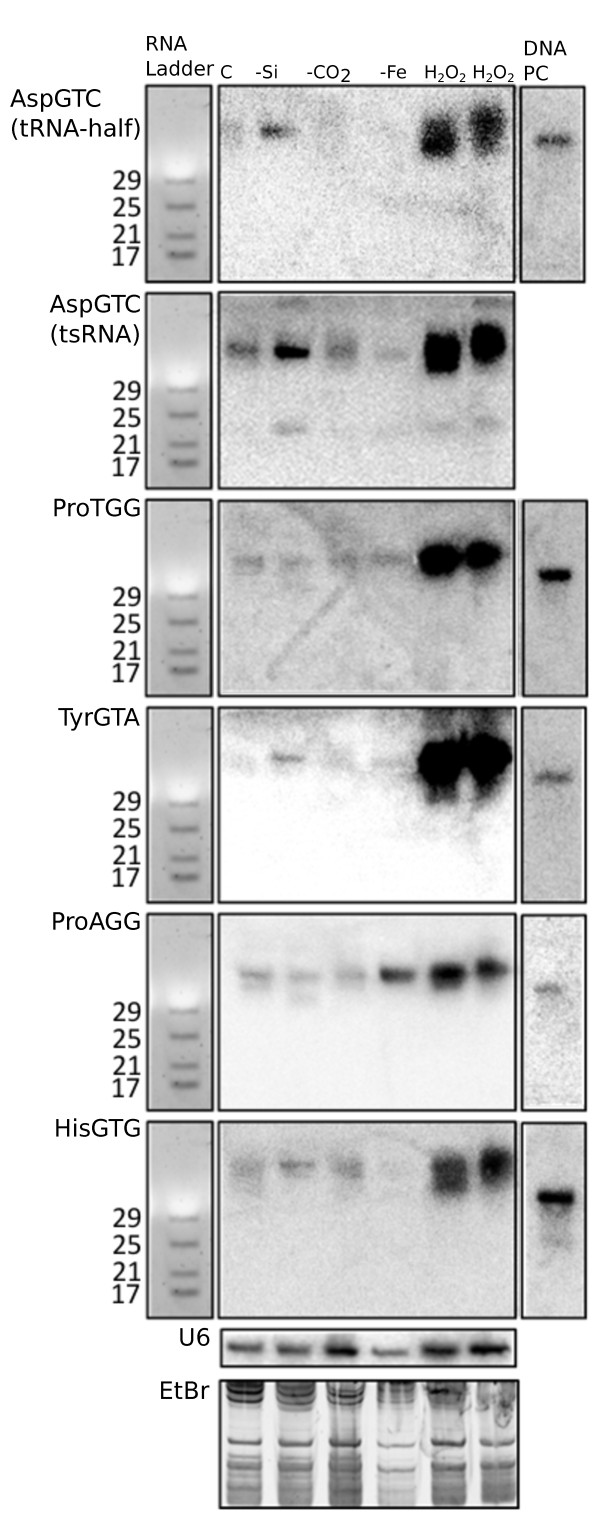
**Northern blot of tRNA-derived sRNAs in** ***T. pseudonana*** **under abiotic stress.** Northern blots for six tRNA-derived sRNAs using RNA from *T. pseudonana* under different growth conditions (C = control growth; -Si = silicate starvation; -CO_2_ = alkaline pH; -Fe = iron limitation; H_2_O_2_ = growth under 20 μM H_2_O_2_, two biological replicate experiments are shown for H_2_O_2_ treatments). ZR small RNA ladder (left), U6 probe hybridisation and EtBr gel (bottom) are provided for band sizing and to show equal loading of the above RNAs. A DNA blot (right) is provided as positive control, and consists of a DNA oligonucleotide with sequence identical to the target sRNA.

In order to test if the tRNA-derived sRNAs found in *T. pseudonana* may be involved in the response to abiotic stress, we analysed their accumulation level by Northern blots using RNA from *T. pseudonana* grown under different environmental conditions (control growth conditions, oxidative stress, silicon- and iron- deficiency as well as alkaline pH) (Figure 5). All of them were strongly up-regulated under oxidative stress (20 μM H_2_O_2_). Also, some of the tRNA-derived sRNAs were up-regulated under other specific stress conditions. ProAGG derived sRNAs showed up-regulation specifically under iron limitation and sRNAs produced from AspGTC (both tRNA-half and tsRNA) were upregulated under silicon starvation.

## Discussion

### Are there canonical micro RNAs in diatoms?

It is generally accepted that sequencing the sRNA transcriptome and using computational methods only to predict miRNA is not rigorous enough to obtain evidence for the existence of miRNAs. We were not able to validate any of the predicted miRNAs from either the study by Norden-Krichmar *et al*. [27] or our own sequencing data by Northern blot experiments, other than a few sequences (Fc-3 and Fc-4) that mapped to tRNA genes. Additionally, we found only one previously predicted *T. pseudonana* miRNA [27] in our libraries (labelled “921_306_230_F3!AR2_G31013_21nts_x451” in [27]). However, this was also found to be part of a tRNA transcript, which demonstrates that ncRNAs such as tRNAs can be false positives in computational miRNA predictions. It is always very difficult to prove the absence of a sequence, but based on our comprehensive approach including experimental validation, the existence of canonical miRNAs as we know them from plants and animals is very unlikely in *T. pseudonana*. In support of this finding is the fact that *T. pseudonana* does not have a canonical Dicer protein either [37]. The putative Dicer protein in *T. pseudonana* only possesses two RNAse III domains, whereas canonical Dicer proteins possess an amino-terminal DEADc/HELICASEc domain, followed by a double-stranded RNA-binding domain (previously referred to as DUF283), a PAZ domain, two RNase III domains and another type of double-stranded RNA-binding domain (dsRBD) (Additional file 1: Figure S5). Dicer-like proteins with two RNaseIII domains have been nevertheless reported to successfully process double-stranded RNA into sRNAs [38]. In contrast, we found that the one candidate Dicer-like protein in the *F. cylindrus* genome (protein ID 242149) appears to have evolved from the fusion of two proteins. The N-terminus of this candidate contains DNA methyltransferase domain, followed by DEADc/HELICASEc domain, double-stranded RNA-binding domain, and two RNase III domains (Additional file 1: Figure S5). It is therefore tempting to speculate that *F. cylindrus* Dicer-like protein can be part of a ribonucleoprotein complex causing methylation of genomic DNA. In addition, phylogenetic reconstruction indicated that the DNMT domain found in this protein is most closely related to DNMT1/Dim-2 subtype that is absent from *P. tricornutum* and *T. pseudonana* [39] but present in *T. oceanica*. Furthermore, a phylogenetic analysis of *T. pseudonana* and *P. tricornutum* Argonaute proteins revealed that they are only distantly related to canonical Argonaute proteins with experimentally validated functions, suggesting functional specialization [37]. Thus, it seems that Dicer and Argonaute proteins in *T. pseudonana* fulfil different functions and hence do not lead to the production of miRNAs unlike their paralogous proteins in most animals and plants.

In summary, our results highlight the need for careful interpretation of miRNA predictions and thorough experimental validation, since other ncRNAs share similar expression and folding characteristics.

### Repeat associated short RNAs in diatoms

The repeat content in *F. cylindrus* (23.6%) is much higher than in *T. pseudonana* (6.5%). However, significantly more sRNAs in *T. pseudonana* (561,235 sequences, 1.9% of mapped sequences) matched to repeats than in *F. cylindrus* (146,818 sequences, 1.6% of mapped sequences) (Additional file 3). A recent study on whole-genome DNA methylation in *P. tricornutum* [12] revealed that 39% of all repeat-rich regions in the genome of this diatom are methylated, and *P. tricornutum* also has about 17% repeats in its genome. The sequence bias towards uracil at the 5’ end of the repeat-associated sRNAs in *T. pseudonana* indicates that they are either produced in a similar way or they are in complex with the same protein once they are produced. Although *T. pseudonana*, *P. tricornutum* and *F. cylindrus* have non-canonical Dicer proteins encoded in their genomes, the first two diatom species seem to have their repeat-associated regions under stronger control by sRNAs than *F. cylindrus.* Repeat-associated sRNAs in *F. cylindrus* did not have a 5’-prime nucleotide bias and did not match to as many repeats as in *T. pseudonana*. If repeat-associated regions in *F. cylindrus* are under weaker control by sRNAs, we could be seeing the effect of a mechanism to adapt to the harsh conditions of the Southern Ocean, where the algae have to cope with low temperatures, strong seasonality and nutrient limitation, hence diversifying gene content could confer an evolutionary advantage.

### Transfer RNA derived sRNAs in diatoms and coccolithophores

Our data on tRNA-derived sRNAs showed the first experimental evidence that diatoms and coccolithophores produce non-coding sRNAs that were under environmental control. This knowledge has significant implications for our understanding on how marine microalgae, which contribute to at least 25% of global CO_2_ fixation [1], cope with environmental changes including nutrient limitations (e.g. iron, silicate, nitrate), changing CO_2_ concentrations and oxidative stress. In both diatom species, tRNA-derived sRNAs represented a large fraction of all sequenced short RNAs, especially in *F. cylindrus* which had 20.7% of all short RNAs derived from tRNAs. sRNAs derived from tRNAs are considered to be produced mostly under stress conditions in eukaryotes and have been shown to regulate translation of mRNA in these organisms [21]. However there are exceptions as some specific tRNA-derived sRNAs are not produced under stress in humans for instance [21]. Interestingly, neither of the diatom species were grown under any kind of stress condition for sequencing the short RNA transcriptomes, yet they produced high level of tRNA-derived sRNAs that became even more abundant under stress conditions as we showed by Northern blots (Figure 5).

Cleavage of tRNAs is considered to have evolved very early in evolution as tRNA-derived sRNAs have been found in all organisms so far including Bacteria and Archaea [22, 40]. In organisms with a longstanding evolutionary trajectory such as bacteria, fungi and excavata (e.g. Trypanosoma), tRNA-derived sRNAs play important roles for post-transcriptional control of gene expression or regulation of translation [40, 41]. Many of these organisms (e.g. Trypanosoma) lack the canonical RNAi machinery and therefore could use these tRNA-derived sRNAs as players of alternative miRNA/siRNA functions. Diatoms and coccolithophores radiated much earlier than most organisms that possess canonical miRNAs/siRNAs such as plants and animals. This would explain why we were not able to identify canonical components of RNAi machinery in diatoms and coccolithophores either. Thus, it is likely that both microalgal groups still use cleavage of tRNAs as a mechanism to post-transcriptionally control gene expression or translation as shown in Trypanosoma, which also has a longstanding evolutionary trajectory. Thus, regulation of translation seems to play a key role in these diatom species and might be orchestrated by tsRNAs.

Diatoms and most likely also coccolithophores clearly seem to use tRNA-derived sRNAs to cope with acclimation to environmental conditions. The stress experiments with *T. pseudonana* reveal that this diatom seems to have stress-specific tRNA-derived sRNAs (e.g. oxidative stress), which indicates the presence of specific regulatory mechanisms to cope with these stresses. Differential expression of tRNA-derived sRNAs under different concentrations of silicate in the growth medium even suggests that tRNA-derived sRNAs might be involved in the regulation of the silica cell wall, the most unique feature of diatoms and key to their success in the oceans [3].

In addition to responses that were specific to different stresses, all tested tRNA-derived sRNAs in *T. pseudonana* were up-regulated under oxidative stress imposed by the addition of H_2_O_2_. This is a common response of many eukaryotic organisms such as yeast, mammalian cells, and plants [21]. Interestingly, the sub-lethal dose of 20 μM H_2_O_2_ did not affect the growth rate but the photosynthetic quantum yield, which indicates a stress condition (Additional file 1: Figure S6). Thus, it seems unlikely that the induction of tRNA-derived sRNAs induced a general down-regulation of translation as the cells would have not been able to continue to grow at the same rate as before (Additional file 1: Figure S6) although we cannot rule out the possibility of the down-regulation of specific proteins.

## Conclusions

This study provides the first experimental evidence for the existence of small non-coding RNAs in marine microalgae that contribute to at least 25% of global carbon fixation and thus represent key organisms that drive biogeochemical cycles on Earth [1]. There is no conclusive evidence of canonical miRNAs as known from plants and animals. However, besides repeat-associated sRNAs, the group of tRNA-derived sRNAs seems to be very prominent in diatoms and coccolithophores and maybe used for acclimation to environmental conditions.

## Methods

### Growth conditions

Axenic *Thalassiosira pseudonana* clone CCMP1335, axenic *Phaeodactylum tricornutum* clone CCMP2561 and axenic *Fragilariopsis cylindrus* clone CCMP1102 for which whole-genome sequences are available were used for experiments in this study. Furthermore, axenic *Emiliania huxleyi* clone RCC1217 was also used. Ten litre cultures of *T. pseudonana*, *P. tricornutum*, *F. cylindrus and E. huxleyi* were grown under optimum growth conditions for each of the species (*T. pseudonana, P. tricornutum and E. huxleyi*: 20°C, 100 μmol photons m^-2^ s^-1^ (24 hours light); *F. cylindrus*: 4°C, 50 μmol photons m^-2^ s^-1^ (24 hours light); nutrient replete F/2 medium; cells were harvested in the middle of exponential growth phase) to obtain RNA for either Illumina sequencing (*T. pseudonana and F. cylindrus*) or Northern Blots (*T. pseudonana, P. tricornutum, F. cylindrus, E. huxleyi*). Total RNA from previous growth experiments with *T. pseudonana* [11] was used for differential expression analysis of sRNAs under abiotic stress. Hydrogen peroxide experiments were conducted with *T. pseudonana* cultures (two biological replicates) at 20°C and 100 μmol photons m^-2^ s^-1^ (24 hours light) in nutrient replete F/2 medium. Hydrogen peroxide was added to both cultures in the middle of the exponential growth phase to obtain a final concentration of 20 μM (Additional file 1: Figure S6). Cells were harvested for RNA extraction 24 hours later.

### RNA extraction

Cells were washed from the filter using 1 ml lysis buffer from mirVana™ kit (Ambion) and bead beating for 60 sec using a Mini beadbeater (Biospec) to break the cells and release the nucleic acid from the samples. The samples were then centrifuged at 10,000 × g for 1 min and the supernatants (750 μl) transferred to a fresh 1.5 ml-microtube. 200 μl additional lysis buffer were added to the filter, bead beating again and centrifuged. The protocol was finished according to the manufacturer’s recommendations. RNA integrity and purity were assessed in 1% (w/v) agarose gel stained with ethidium bromide and on a Nanodrop ND-1000 (Thermo Scientific, DE, USA), respectively. Ratios of Abs 260/280 and Abs 260/230 were above 2.0 for all RNA samples.

### sRNA libraries

The first set of sRNA libraries was done as described before [30] using total RNA from *T. pseudonana* grown in normal conditions, iron- and silicon- limitation as well as alkaline pH [11]. Briefly, the RNA was run in a 15% polyacrylamide gel, and the area corresponding to 19–24 nt length was excised and eluted. The RNA was ligated to 5’- and 3’-RNA adaptors, and after each ligation the products were gel-purified to eliminate non-ligated adaptors. RNA was converted to DNA by RT-PCR, and sequenced (36 cycles) using an Illumina Genome Analyzer II at BaseClear (Leiden, The Netherlands).

Total RNA from *T. pseudonana* and *F. cylindrus* was used for the second set of libraries, using the small RNA v1.5 kit from Illumina according to manufacturer’s recommendations. With this protocol it is not necessary to gel-purify the ligation products after each ligation. RNA was ligated to the adaptors, reverse transcribed, PCR amplified and purified from a polyacrylamide gel. cDNA libraries were sequenced (42 cycles) using an Illumina Genome Analyzer II at TGAC (Norwich, UK).

### Northern blotting

Five μg of total RNA was used for sRNA Northern blot analysis as described previously [30]. Briefly, RNA was separated in a 15% denaturing polyacrylamide gel, blotted to Hybond NX membranes (Amersham) and chemically crosslinked. Expression of small RNAs was assessed by hybridisation to a gamma [P^32^]-labelled (Perkin Elmer, UK) nucleic acid oligonucleotide probe. EtBr staining and U6 northern blotting were used to assess equal loading. ZR small RNA ladder (Zymoresearch) was used to size the bands. DNA oligonucleotides with sequence identical to the target sRNA were used as positive controls and, when possible, the primers were loaded in the gel along with the samples. If membranes were re-probed, they were stripped and exposed for several days to check efficient probe removal before re-probing. Positive control and probe sequences are provided in Additional file 4.

### Bioinformatics analysis

3’ adapters were trimmed from the sequences by matching the first 6 nucleotides without mismatches. Sequences where no adapter was matched were discarded. After adapter trimming, 63,419,714 and 5,437,551 redundant sequences were left for *T. pseudonana* and *F. cylindrus* respectively. Genomes for the two diatoms were obtained from http://genome.jgi-psf.org/Thaps3 and http://genome.jgi-psf.org/Fracy1/. The latest releases were used, v3.0 for *T. pseudonana* and v1.0 for *F. cylindrus*. Reads were mapped to their respective genomes using PatMaN [42] with no mismatches or gaps. After mapping, we found two very abundant sequences (one in *T. pseudonana* and one in *F.* cylindrus) that mapped to rRNA loci. These were not included in the final size class distributions to avoid skewing the data.

To determine sources of sRNAs, an annotation pipeline was built using several alignment and prediction tools. Since there was no information on diatom tRNAs, we generated a list of tRNA candidate sequences by running tRNAscan-SE [43] (version 1.3.1), using the default parameters on both genomes. The sequences were post-transcriptionally modified by removing intron sequences labelled by tRNAscan-SE and adding a ‘CCA’ motif to the 3’ end of each sequence (the resulting predictions are listed in Additional file 5). This allowed us to accurately align sequences to the reference tRNAs without using mismatches, since the tRNAs are post-transcriptionally modified within the cell (Additional file 5). We found 71 tRNAs in *T. pseudonana* and 163 tRNAs in *F. cylindrus* (Additional file 6). We mapped reads to the modified tRNA sequences using PatMaN with no mismatches, resulting in 887,404 redundant sequences in *T. pseudonana* and 1,865,136 redundant sequences in *F. cylindrus* mapping to tRNA sequences. We also used RNAmmer 1.2 [44] to predict rRNA sequences in both diatoms and annotated reads as rRNA-derived if they overlapped these predictions. The remaining annotations were annotated by finding reads that overlapped the set of repetitive elements in both diatoms, provided by Florian Maumus (INRA, Versailles, France), and also the set of predicted genes available for each genome. Finally, to identify any sequences that mapped to remaining potential unannotated ncRNAs in the diatom genomes, the sRNAs were searched against Rfam [45] using BLASTN [46] with the window parameter set to 6, DUST filtering turned off, and an e-value cut-off of 10. Hits were accepted if there was an 80% identity along the whole length of the sequence.

We used both miRCat [32] and miRDeep2 [33] to predict potential miRNA candidates in both diatoms. We ran miRCat once using default animal-based parameters and once using default plant-based parameters to broaden the search space that diatom miRNAs may be residing in.

The data sets supporting the results of this article are available in the GEO repository at NCBI under the accession name GSE57987.

## Electronic supplementary material

Additional file 1: Figure S1: PAGE of sRNA libraries from *F. cylindrus* and *T. pseudonana.* 15% Polyacrylamide gel stained with EtBr showing cDNA libraries of sRNAs obtained from RNA from *F. cylindrus* (Fc) and *T. pseudonana* (Tp). RNA from tomato leaf and fruit, and a DNA marker are provided for size comparison and quantification purposes respectively. **Figure S2** - Venn diagram of miRNA predictions*.* Venn diagram depicting the number of predictions by miRNA prediction tools miRCat with plant parameters, miRCat with animal parameters [32], and miRDeep2 [33]. **Figure S3** - Size class distribution of sRNAs mapping to repetitive elements. Redundant counts for sRNAs that mapped only to repetitive elements are shown for each diatom with colours based on the 5’ most nucleotide of sequences. **Figure S4** - tRNA-derived sRNA summary. a) Correlation of total tRNA abundances for each tRNA between diatoms. b) Top cleavage sites for the most abundant tRNAs in each diatom. Arrows indicate where the cleavage happens in a particular diatom, and the percentages listed are out of all tRNA derived sRNAs. c and d) relationship between tRNA abundance and copy number in each diatom. **Figure S5** - Schematics indicating domains for possible Dicer candidates. Bioinformatic prediction of protein domains in putative Dicer-like proteins in *T. pseudonana* (Tp-Dcl1) and *F. cylindrus* (Fc_Dcl1) compared to *Homo sapiens* Dicer protein (Hs_Dcr). DEXDc/HELc: DEADc/HELICASEc domain, dsRNA_bind: double-stranded RNA-binding domain, DSRM: double-stranded RNA-binding domain, DNMT: DNA methyltransferase domain. The prediction was done using RPS BLAST against the Conserved Domain Database, (online at http://www.ncbi.nlm.nih.gov/Structure/cdd/wrpsb.cgi). **Figure S6** - Hydrogen peroxide experiment with *T. pseudonana. T. pseudonana* growth conditions (number of cells, photosynthetic activity). Arrows indicate the points in time when hydrogen peroxide (20 μM) was added and when the culture was harvest for two biological replicates. (PDF 626 KB)

Additional file 2: **Predicted miRNA annotations.** A table of all possible alignments of reads that were predicted as mature miRNAs, including information on annotation that overlapped with the read. (CSV 3 KB)

Additional file 3: **Feature count table.** Summary of redundant read counts split into the different feature categories and the percentages that these features make up of the total read count. (XLSX 12 KB)

Additional file 4: **Probe sequences.** Sequences of oligonucleotides used as probes or positive controls for the Northern blots shown in this paper. (CSV 1 KB)

Additional file 5: **tRNAscan predicted tRNAs.** Output of trnascan for each organism it was run on. These results were further processed to derive the mature sequence and structures (addition of CCA motif, removal of introns), which are also listed. (CSV 87 KB)

Additional file 6: **tRNA summary.** Summary of the tRNAs predicted by tRNA scan, the number of transcripts found in both diatoms for each type of tRNA, and a weighted count of the number of sequences that align to the mature tRNA sequences. The count is weighted by dividing the count of a sequence by the number of times it mapped to the tRNA references. (CSV 1 KB)
