# Supplementary material for: Global discovery and characterization of small non-coding RNAs in marine microalgae
Source: BMC Genomics. 2014 Aug 20;15(1):697. doi: 10.1186/1471-2164-15-697 (PMC4156623; doi:10.1186/1471-2164-15-697)
Supplement: Supplementary file 1 — Additional file 1: Figure S1: PAGE of sRNA libraries from F. cylindrus and T. pseudonana. 15% Polyacrylamide gel stained with EtBr showing cDNA libraries of sRNAs obtained from RNA from F. cylindrus (Fc) and T. pseudonana (Tp). RNA from tomato leaf and fruit, and a DNA marker are provided for size comparison and quantification purposes respectively. Figure S2 - Venn diagram of miRNA predictions. Venn diagram depicting the number of predictions by miRNA prediction tools miRCat with plant parameters, miRCat with animal parameters [32], and miRDeep2 [33]. Figure S3 - Size class distribution of sRNAs mapping to repetitive elements. Redundant counts for sRNAs that mapped only to repetitive elements are shown for each diatom with colours based on the 5’ most nucleotide of sequences. Figure S4 - tRNA-derived sRNA summary. a) Correlation of total tRNA abundances for each tRNA between diatoms. b) Top cleavage sites for the most abundant tRNAs in each diatom. Arrows indicate where the cleavage happens in a particular diatom, and the percentages listed are out of all tRNA derived sRNAs. c and d) relationship between tRNA abundance and copy number in each diatom. Figure S5 - Schematics indicating domains for possible Dicer candidates. Bioinformatic prediction of protein domains in putative Dicer-like proteins in T. pseudonana (Tp-Dcl1) and F. cylindrus (Fc_Dcl1) compared to Homo sapiens Dicer protein (Hs_Dcr). DEXDc/HELc: DEADc/HELICASEc domain, dsRNA_bind: double-stranded RNA-binding domain, DSRM: double-stranded RNA-binding domain, DNMT: DNA methyltransferase domain. The prediction was done using RPS BLAST against the Conserved Domain Database, (online at http://www.ncbi.nlm.nih.gov/Structure/cdd/wrpsb.cgi). Figure S6 - Hydrogen peroxide experiment with T. pseudonana. T. pseudonana growth conditions (number of cells, photosynthetic activity). Arrows indicate the points in time when hydrogen peroxide (20 μM) was added and when the culture was harvest for two biological replica [file 12864_2014_6400_MOESM1_ESM.pdf]

Additional File 1 – Supplementary figures (supplementary_figures.pdf)

Figure S1 – PAGE of sRNA libraries from *F. cylindrus* and *T. pseudonana*

15% Polyacrylamide gel stained with EtBr showing cDNA libraries of sRNAs obtained from RNA from *F. cylindrus* and *T. pseudonana*. RNA from tomato leaf and fruit for size comparison purposes. DNA marker for quantification purposes.

Figure S2 - Venn diagram of miRNA predictions

Venn diagram depicting the number of predictions by miRNA prediction tools: miRCat (plant parameters), miRCat (animal parameters), and miRDeep2

Figure S3 - Size class distribution of sRNAs mapping to repetitive elements.

Redundant counts for sRNAs that mapped only to repetitive elements are shown for each diatom with colours based on the five prime most nucleotide of sequences.

Figure S4 - tRNA-derived sRNA summary

a) Correlation of total tRNA abundances for each tRNA between diatoms. b) Top cleavage sites for the most abundant tRNAs in each diatom. Arrows indicate where the cleavage happens in a particular diatom, and the percentages listed are out of all tRNA derived sRNAs. c)/d) relationship between tRNA abundance and copy number in each diatom.

Figure S5 - Schematics indicating domains for possible dicer candidates.

Bioinformatic prediction of protein domains in putative Dicer-like proteins in *T. pseudonana* (Tp-Dcl1) and *F. cylindrus* (Fc_Dcl1) compared to *Homo sapiens* Dicer protein (Hs_Dcr). DEXDc/HELc: DEADc/HELICASEc domain, dsRNA_bind: double-stranded RNA-binding domain, DSRM: double-stranded RNA-binding domain, DNMT: DNA methyltransferase domain. The prediction was done using RPS BLAST against the Conserved Domain Database, (online at http://www.ncbi.nlm.nih.gov/Structure/cdd/wrpsb.cgi).

Figure S6 - Hydrogen peroxide experiment with *T. pseudonana.*

T. pseudonana growth conditions (number of cells, photosynthetic activity). Arrows indicate the points in time when hydrogen peroxide (20 µM) was added and when the culture was harvest for two biological replicates.

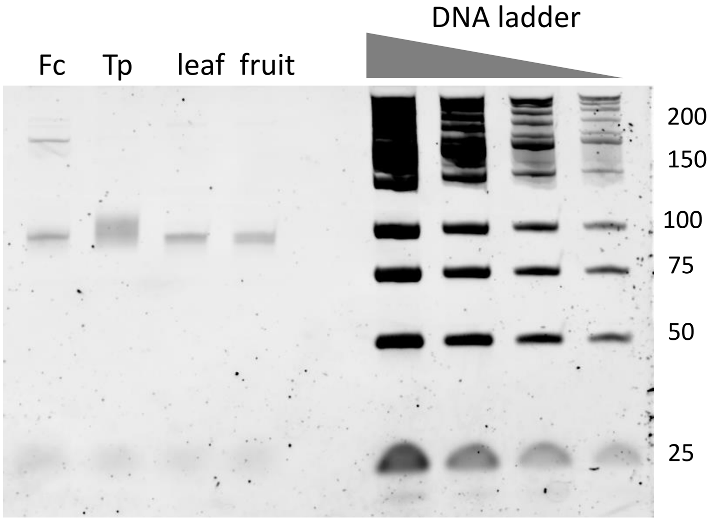

Figure S1

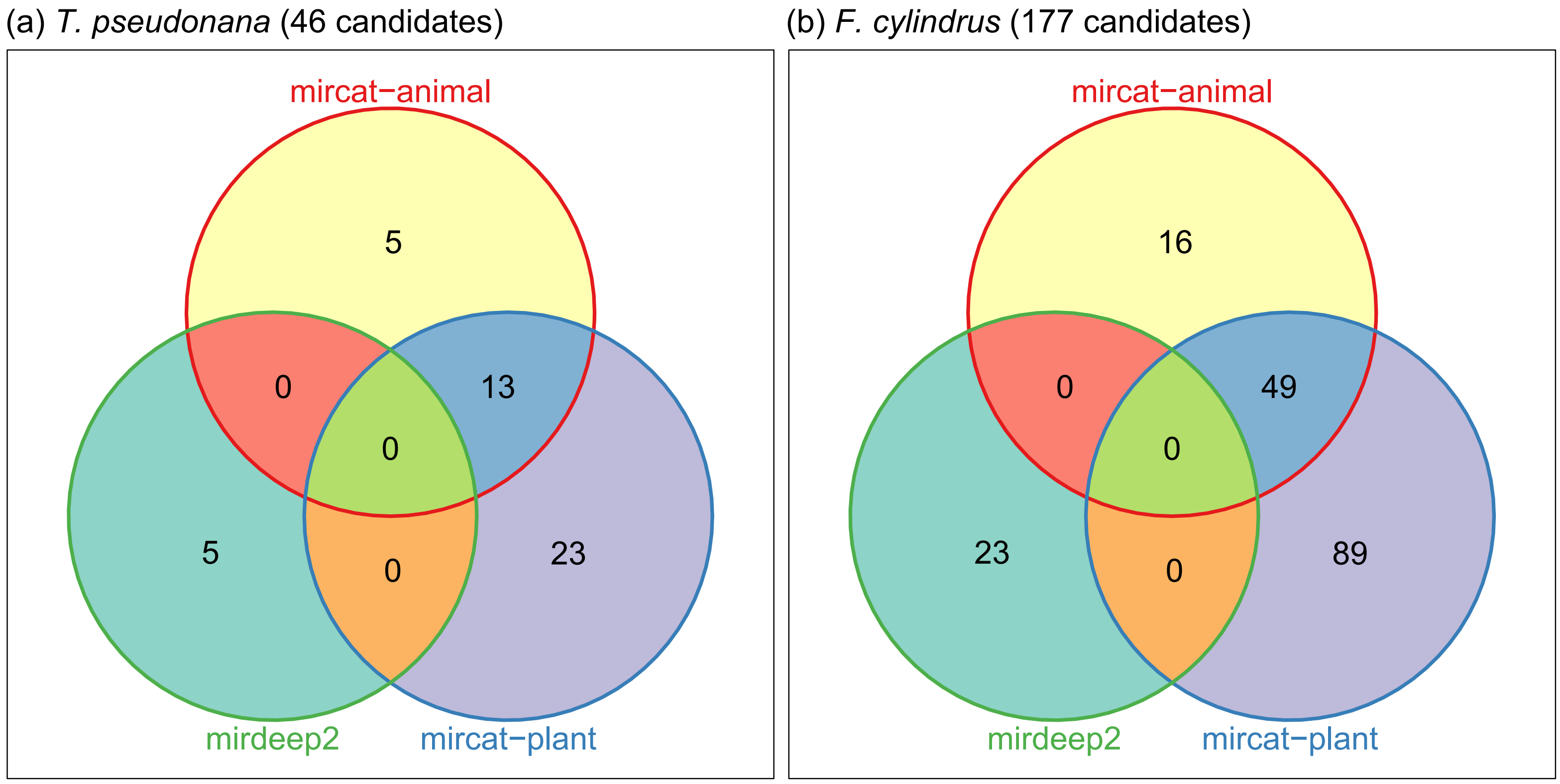
(a) T. pseudonana (46 candidates)
mircat−animal
5
0
13
0
5
0
23
mirdeep2
mircat−plant
(b) F. cylindrus (177 candidates)
mircat−animal
16
0
49
0
23
0
89
mirdeep2
mircat−plant

Figure S2

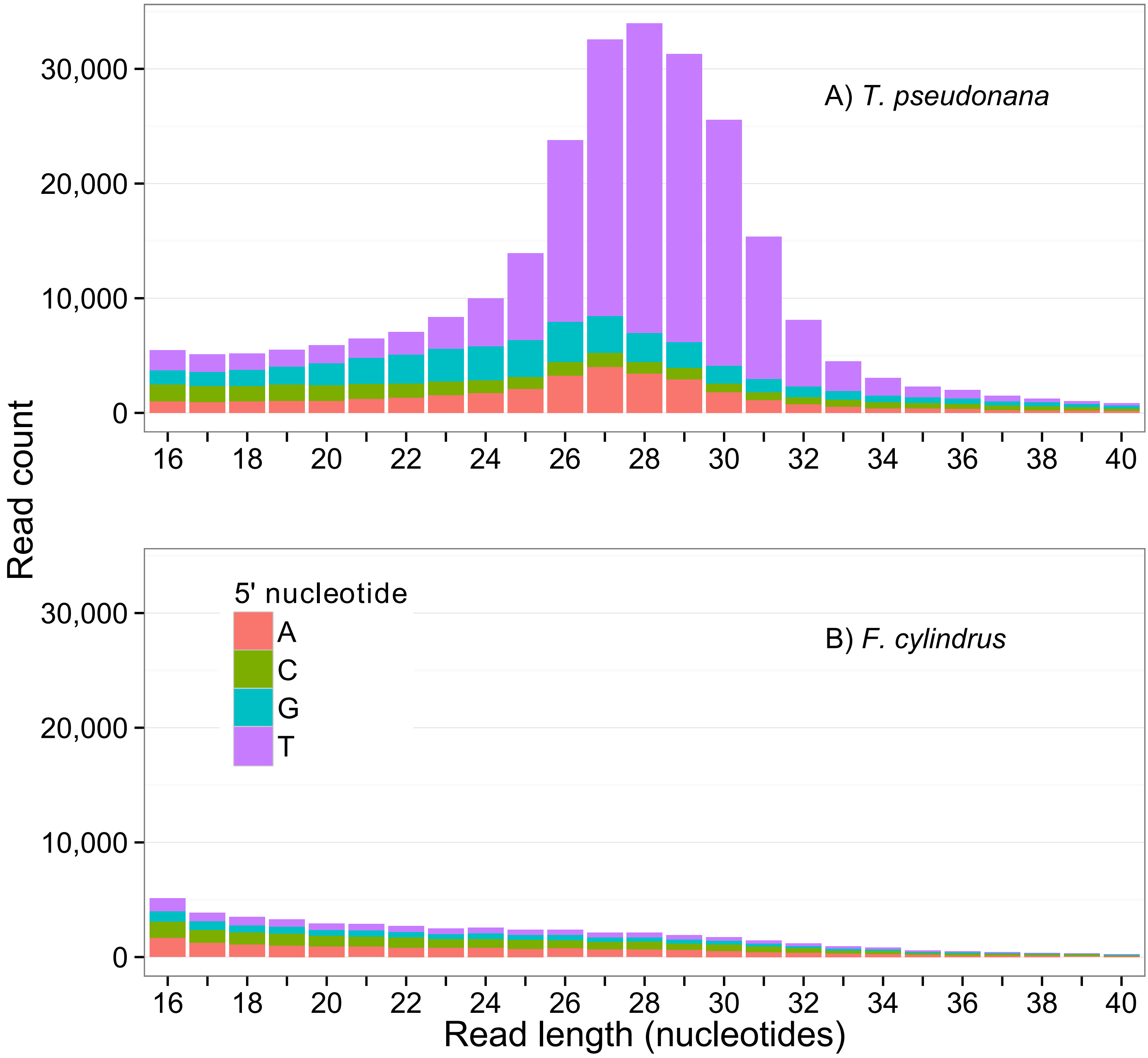
Read count
30,000
20,000
10,000
0
A) T. pseudonana
5' nucleotide
A
C
G
T
B) F. cylindrus
16
18
20
22
24
26
28
30
32
34
36
38
40
Read length (nucleotides)

Figure S3

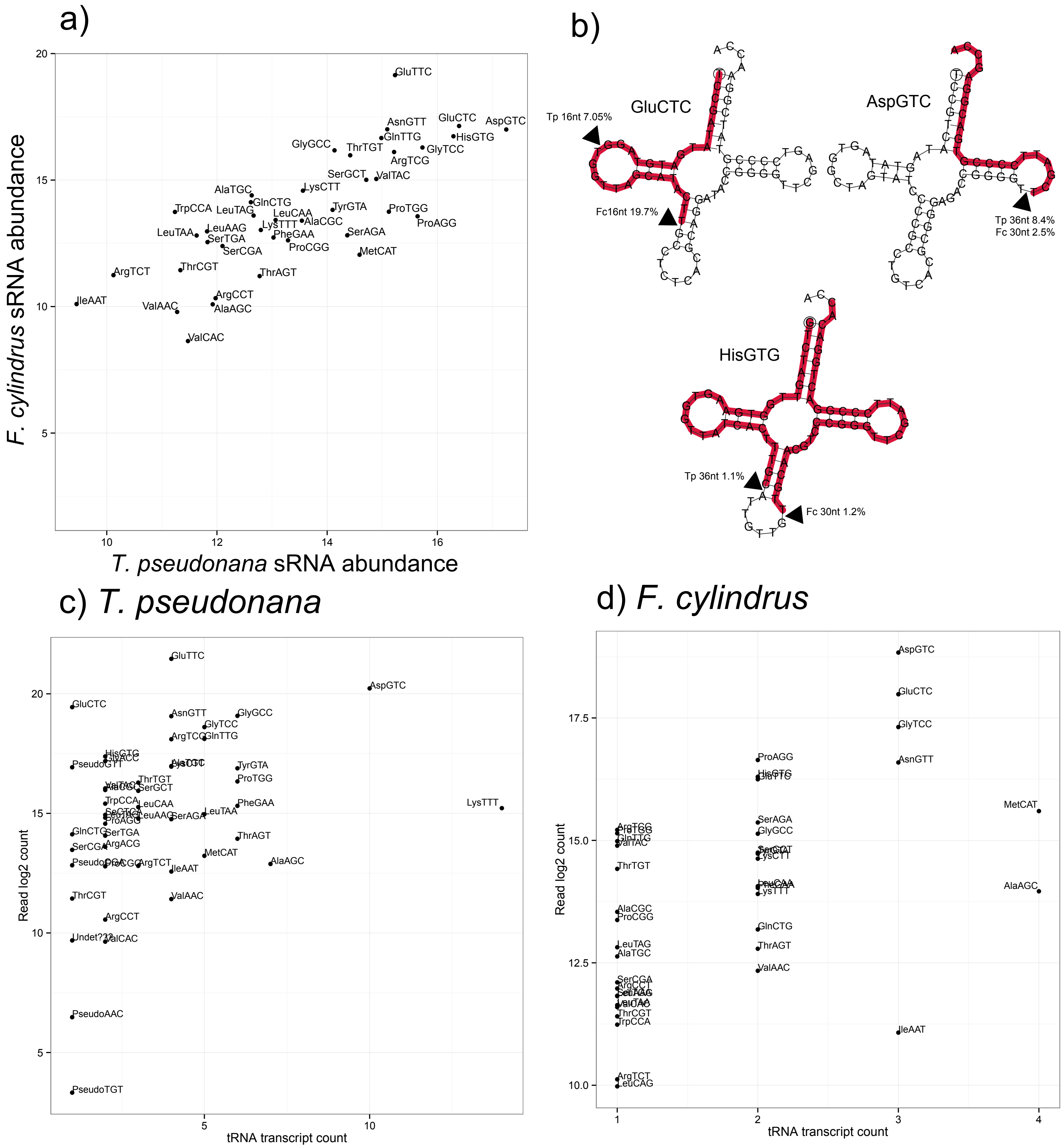
a)
F. cylindrus sRNA abundance
T. pseudonana sRNA abundance
GluTTC
AsnGTT
GluCTC
AspGTC
GlnTTG
HisGTG
GlyGCC
ThrTGT
GlyTCC
ArgTCG
SerGCT
ValTAC
AlaTGC
LysCTT
GlnCTG
TrpCCA
LeuTAG
LeuCAA
TyrGTA
ProTGG
ProAGG
LeuAAG
LysTTT
AlaCGC
LeuTAA
SerTGA
PheGAA
SerAGA
SerCGA
ProCGG
MetCAT
ArgTCT
ThrCGT
ThrAGT
IleAAT
ValAAC
ArgCCT
AlaAGC
ValCAC
b)
GluCTC
AspGTC
HisGTG
Tp 16nt 7.05%
Fc16nt 19.7%
Tp 36nt 8.4%
Fc 30nt 2.5%
Tp 36nt 1.1%
Fc 30nt 1.2%
c) T. pseudonana
Read log2 count
tRNA transcript count
GluTTC
AspGTC
GluCTC
AsnGTT
GlyGCC
GlyTCC
PseudoGTT
TyrGTA
ProTGG
ThrTGT
SerGCT
TrpCCA
LeuCAA
ProAGG
LysTTT
PheGAA
LeuTAA
SerAGA
GlnCTG
SerTGA
ArgACG
SerCGA
ThrAGT
MetCAT
ArgTCT
AlaAGC
IleAAT
ThrCGT
ValAAC
ArgCCT
ValCAC
PseudoAAC
PseudoTGT
d) F. cylindrus
Read log2 count
tRNA transcript count
AspGTC
GluCTC
GlyTCC
AsnGTT
ProAGG
MetCAT
SerAGA
GlyGCC
ValTAC
ThrTGT
AlaGC
AlaCGC
ProCGG
GlnCTG
ThrAGT
LeuTAG
AlaTGC
ValAAC
SerCGA
ThrCGT
TrpCCA
IleAAT
ArgTCT
LeuCAG

Figure S4

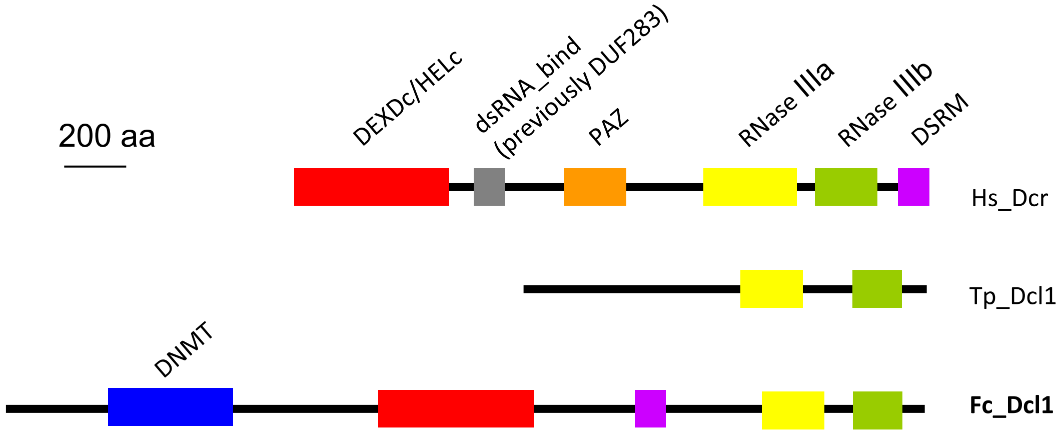
200 aa
DEXDc/HELc
dsRNA_bind
(previously DUF283)
PAZ
RNase IIIa
RNase IIIb
DSRM
Hs_Dcr
Tp_Dcl1
DNMT
Fc_Dcl1

Figure S5

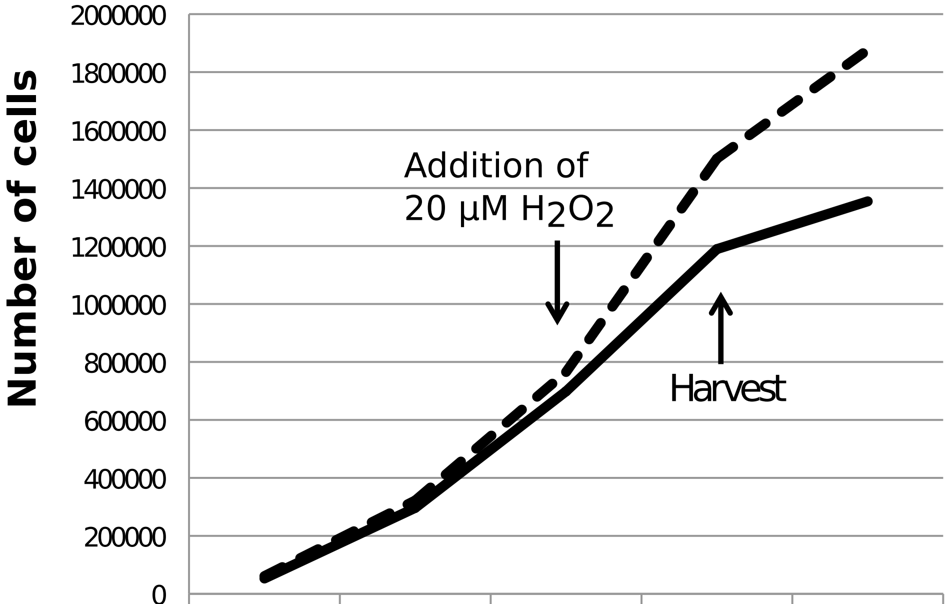

	1	2	3	4	5
Replicate 1	52920	296700	698700	1189300	1354100
Replicate 2	61200	322000	765400	1501600	1876900

Time (days)

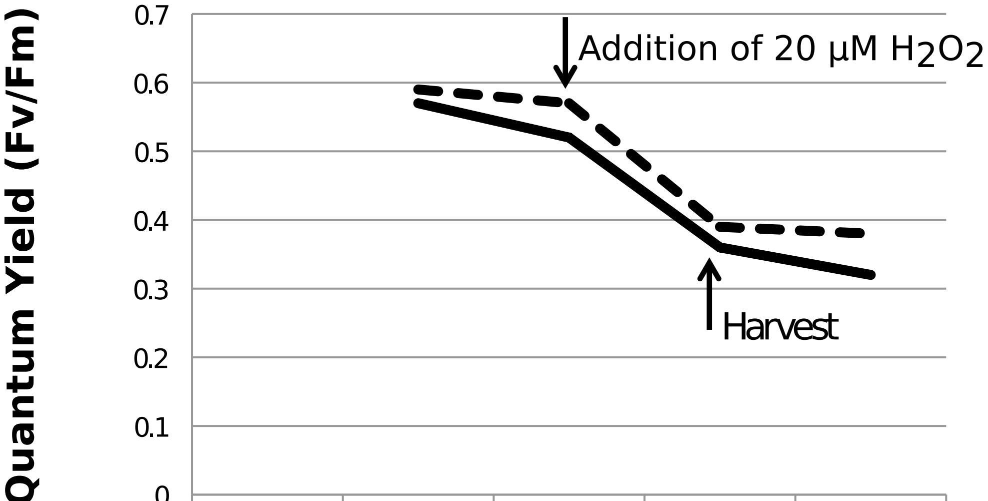

	1	2	3	4	5
Replicate 1		0.57	0.52	0.36	0.32
Replicate 2		0.59	0.57	0.39	0.38

Time (days)

Figure S6
